# DNA methylation screening after roux-en Y gastric bypass reveals the epigenetic signature stems from genes related to the surgery per se

**DOI:** 10.1186/s12920-019-0522-7

**Published:** 2019-05-27

**Authors:** C. F. Nicoletti, M. A. S. Pinhel, A. Diaz-Lagares, F. F. Casanueva, A. Jácome, V. C. Pinhanelli, B. A. P. de Oliveira, A. B. Crujeiras, C. B. Nonino

**Affiliations:** 1 Laboratory of Nutrigenomics Studies, Department of Internal Medicine, Ribeirão Preto Medical School, University of Sao Paulo, Sao Paulo, Brazil; 2 Cancer Epigenomics, Translational Medical Oncology (Oncomet), Health Research Institute of Santiago (IDIS), University Clinical Hospital of Santiago (CHUS/SERGAS), Santiago de Compostela, Spain; 3 Centro de Investigacion Biomedica en Red Cancer (CIBERONC), Madrid, Spain; 4 Epigenomics in Endocrinology and Nutrition, Health Research Institute of Santiago (IDIS), University Clinical Hospital of Santiago (CHUS/SERGAS) and Santiago de Compostela University (USC), Santiago de Compostela, Spain; 5 CIBER Fisiopatologia de la Obesidad y Nutricion (CIBERobn), Madrid, Spain; 6 Department of Mathematics, MODES group, CITIC, Universidade da Coruña, Faculty of Science, A Coruña, Spain

**Keywords:** Obesity, Bariatric surgery, Epigenetics, DNA methylation, Pathway, Weight loss

## Abstract

**Background/objectives:**

Obesity has been associated with gene methylation regulation. Recent studies have shown that epigenetic signature plays a role in metabolic homeostasis after Roux-en Y gastric bypass (RYGB). To conduct a genome-wide epigenetic analysis in peripheral blood to investigate whether epigenetic changes following RYGB stem from weight loss or the surgical procedure per se.

**Subjects/methods:**

By means of the Infinium Human Methylation 450 BeadChip array, global methylation was analyzed in blood of 24 severely obese women before and 6 months after RYGB and in 24 normal-weight women (controls).

**Results:**

In blood cells, nine DMCpG sites showed low methylation levels before surgery, methylation levels increased after RYGB and neared the levels measured in the controls. Additionally, 44 CpG sites associated with the Wnt and p53 signaling pathways were always differently methylated in the severely obese patients as compared to the controls and were not influenced by RYGB. Finally, 1638 CpG sites related to inflammation, angiogenesis, and apoptosis presented distinct methylation in the post-surgery patients as compared to the controls.

**Conclusion:**

Bariatric surgery per se acts on CpGs related to inflammation, angiogenesis, and endothelin-signaling. However, the gene cluster associated with obesity remains unchanged, suggesting that weight loss 6 months after RYGB surgery cannot promote this effect.

**Graphical abstract:**

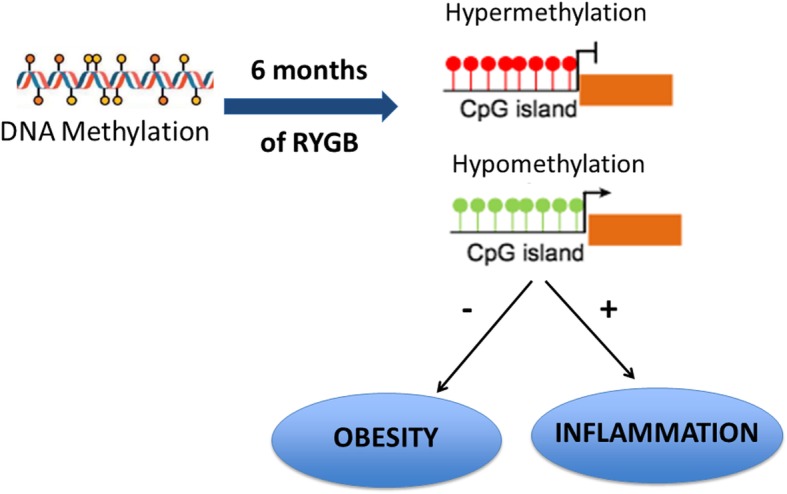

**Electronic supplementary material:**

The online version of this article (10.1186/s12920-019-0522-7) contains supplementary material, which is available to authorized users.

## Background

There has been increasing evidence that obesity is not a simple consequence of unbalanced dietary habits or sedentary behaviors, but a systemic and complex disease resulting from body homeostasis and metabolism dysregulation [1, 2]. In this sense, genetic approaches have demonstrated that obesity could be an inherited disease stemming from genetic background and heritable epigenetic factors [3].

In the context of epigenetics research, DNA methylation has been the most extensively studied phenomenon related to obese and metabolic phenotypes [4]. DNA methylation consists in addition of methyl (−CH3) groups at CpG dinucleotides, which influences gene transcription [5]. Regarding predisposition to metabolic disorders, obesity has been associated with regulation of the methylation of numerous candidate genes [6]. Animal studies have shown that high-fat diet modifies the epigenetics and transcriptional activity of lipid homeostasis-related genes, which contributes to obesity development [7]. On the other hand, obesity-induced inflammation and oxidative stress due to fat accumulation exposes the genome of many tissues to several systemic factors, which can determine the DNA methylation profile [8]. Indeed, this abnormal global epigenetic state drives obesogenic expression patterns [9]. Therefore, alterations in the epigenome may cause molecular changes in pathways that are associated with obesity and may improve metabolic health after therapeutic intervention [10].

Concerning obesity treatments, bariatric surgery is the most effective intervention for severe obese. Roux-en Y gastric bypass (RYGB) is one of the most often performed techniques and corresponds to 43% of all bariatric surgery procedures [11, 12]. Although many mechanisms may contribute to weight loss and metabolic improvement after RYGB (e.g., decreased food ingestion, changes in gut hormones and peptide secretion, and nutrient disabsortion) [13, 14], recent studies have demonstrated that epigenetic signature plays a role in metabolic homeostasis after surgery [15, 16]. Altered methylation of specific DNA sites has been verified after weight loss [16, 17] and bariatric surgery [18].

Knowing whether epigenetic alterations stem from weight and fat loss or the surgical procedure per se is important. Some methylation changes may result from obesity phenotype marks [4]; however, the DNA methylation profile can be a biological effect of calorie restriction [19]. Even after significant weight is lost and comorbidities are improved, patients submitted to RYGB remain with an obese profile at the early postoperative period [20]. Bariatric surgery can change the molecular pathways involved in inflammatory and immunological response, cell differentiation, and oxidative stress regulation [21], so we suggest that the surgical procedure itself may epigenetically modify obesity-independent genes.

In this study, we conduct a genome-wide epigenetic analysis in peripheral blood to investigate the role that RYGB plays in DNA methylation pattern changes. Also, we evaluate whether and to what magnitude DNA methylation profile changes are associated with the molecular pathogenic mechanism of weight loss and/or with the response pathways related to the surgical procedure.

## Methods

### Subjects and phenotypic characteristics

This is a prospective study involving adult female subjects from a mixed ethnicity population assisted at a public health service in Brazil. The subjects were divided into two groups: 1. RYGB Group: 24 severely obese women (Body mass index (BMI) > 35 kg/m^2^, 36.9 ± 10.2 years) submitted to bariatric surgery (RYGB technique), and 2. Control Group or controls: 24 normal-weight women (BMI ranging from 18.5 to 24.9 kg/m2, 36.9 ± 11.8 years). Men were excluded to avoid the possible biases due to the hormonal influences. Subjects belonging to the RYGB Group were selected from the Bariatric Surgery Outpatient Clinic of a university hospital and had no history of diabetes mellitus. Controls had not had any body weight changes in the previous three months. Patients submitted to the modified standard surgical technique (RYGB), who missed the service follow up, who were pregnant or lactating, and who had a history of alcohol or drug abuse were excluded. Participants were informed about the study protocol, and only those who agreed with its implementation were included.

The RYGB Group was evaluated before (baseline) and six months after bariatric surgery, the Control Group was evaluated only once. Anthropometric measurements (weight, height, BMI, and waist circumference (WC)), body composition analysis (fat mass and fat-free mass), and biochemical evaluation (glucose, total cholesterol, HDL cholesterol, LDL cholesterol, and triglycerides) were accomplished as described previously [22].

### DNA methylation analysis

Blood for DNA methylation analysis was collected after fasting, according to standard procedures. DNA was extracted from peripheral blood leukocytes with the GE Health Care kit (Illustra blood genomic Prep Mini Spin kit), according to the supplier’s instructions. DNA fragmentation or RNA contamination was analyzed by 1% agarose gel electrophoresis. DNA (500 ng) bisulfite was converted by using the EZ DNA methylation kit Methylation-Gold (Zymo Research, CA, USA), according to the manufacturer’s instructions, which converted cytosine to uracil.

Methylation was analyzed with the Infinium Human Methylation 450 BeadChip array). High-quality treated DNA was hybridized to the Infinium Human Methylation 450 BeadChips (Illumina) following the Illumina Infinium HD methylation protocol. Beadchips were scanned with the Illumina HiScanSQ system, and image intensities were extracted with the Genome Studio (2011.1) Methylation Module (v1.8.5). Blood samples from each subject were hybridized to the same physical chip to minimize biases.

DNA quality checks, bisulfite modification, hybridization, data normalization, and statistical filter were performed as described elsewhere [23, 24]. The methylation level was expressed as a beta (β) value that was calculated as the intensity of the methylated channel divided by the total intensity (β = Max (SignalB, 0) / (Max (SignalA, 0) + Max (SignalB, 0) + 100)). β values range from 0 (unmethylated) to 1 (fully methylated) and can be broadly interpreted as the percentage of CpG methylation. For genomic regions, methylation was calculated as the mean β for all the probes located within the region annotated by Illumina: TSS200 (TSS - transcription start site), TSS1500, 5′UTR (UTR - untranslated region), 1st Exon, gene body, 3′UTR, and intergenic. In addition, the probes that were considered single nucleotide polymorphisms were filtered out. The final amount of valid CpGs in this study was 476,895.

Differential methylation analyses aimed to evaluate methylation differences between the study groups. To this end, the mean beta variation (Δβ) was calculated for a given CpG site by subtracting the mean beta value from the pool of pre-surgery samples (pre-surgery period) as compared to the pool of samples collected after RYGB (six months after surgery) or from the pool of pre-surgery samples as compared to the pool of samples from the Control Group.

### Statistical analysis

The phenotypic characteristics of the subjects included in the study were expressed as mean values and standard deviation. Data normality was verified by the Shapiro-Wilk test. Paired t test was carried out to compare pre- and post-operative values, and independent t test was used to compare the RYGB Group and the Control Group. Linear regression models were employed to test how the CpG methylation level affected the anthropometric and biochemical characteristics, adjusted for age. Also, Bonferroni’s correction for multiple comparisons was applied. All the analyses were conducted with the Statistical Package software for Social Sciences (SPSS version 20.0, Inc. Chicago, IL), significance was set at *p* < 0.05.

To identify consistent patterns of differentially methylated CpGs, parametric t test was accomplished. Sample size led us to apply t test instead of other methods that can adjust for confounding factors. Values of *p* were adjusted for multiple comparisons by using the false discovery rate procedure described by Benjamini and Hochberg. In this analysis, a false discovery rate below 5% (q value) was considered statistically significant. However, given the sample size, raw *p* values of 0.01 were selected as a less stringent cutoff for differential methylation than q values. Indeed, a threshold for the significant CpG sites based on Δβ with a minimum value of 5% (value greater than 0.05 or less than − 0.05) was applied. Results were quite robust even though only individuals were evaluated in each group. These statistical analyses were performed with R software (version 3.2.0).

By crossing and comparing the differentially methylated CpG sites (DMCpGs) identified in two different periods (before and after RYGB**)** and in two study groups (pre-surgery patients versus controls and post-surgery patients versus controls), a Venn diagram was created (http://bioinfogp.cnb.csic.es/tools/venny/). Hierarchical cluster analysis of the significant CpGs was carried out with Heatmap function and Genome Studio (2011.1).

To gain even better understanding of the biological relevance of the significant associations between DNA methylation and the studied phenotypes, a hypergeometric test was conducted for the biological processes defined by gene ontology (GO) [25]. This evaluation identified the significant over-representation of GO terms in our lists of selected genes with respect to the other for the entire genome. The IDs were loaded and analyzed against the human reference genome by means of a false discovery rate threshold of *p* < 0.05.

## Results

### Phenotypic characteristics

The severely obese patients had higher weight, BMI, WC, percentage of fat mass (%FM), triglycerides, and total cholesterol as well as lower plasma HDL cholesterol than the controls. RYGB significantly reduced weight, BMI, WC, percentage of fat- free mass, %FM, and serum glucose and lipid levels, but it did not change HDL cholesterol. However, the post-surgery patients still presented anthropometric measurements that are characteristic of obesity, but their lipid profiles (except HDL cholesterol) resembled the lipid profiles of the controls (Additional file 1).

### Methylation data

All results are summarized in Additional file 3. After normalization, we found 476,895 final valid CpG sites and pre, posoperative and normal weight results are present at below topics. Raw data is in Additional file 4.

### Identification of different methylated CpGs between groups and surgery times

Comparison between the methylation profiles of the pre-surgery patients and the controls revealed 1074 DMCpG sites that were related to 769 unique genes. Significant differences between the groups ranged from 0.05 to 0.27 (from 5 to 27%). Even though there was no statistical difference between the average DNA methylation levels of the 1074 DMCpG sites (0.42 ± 0.26 versus 0.41 ± 0.28, *p* = 0.168), the majority of the CpG sites (66.2%) showed higher methylation in the pre-surgery patients, especially in the TSS200 region and gene island (Additional file 2).

Additionally, RYGB elicited changes in 666 CpG sites located in 495 unique genes. Significant differences between the pre- and post-surgery periods ranged from 0.05 to 0.10 (from 5 to 10%), and the average DNA methylation levels of all the DMCpG sites increased after surgery (from 0.44 ± 0.13 to 0.49 ± 0.12, *p* < 0.001). These results showed higher methylation of these CpG sites after RYGB.

Comparison between the post-surgery patients and the controls detected 3223 DMCpG sites (2065 unique genes). Significant differences between the groups ranged from 0.05 to 0.31 (from 5 to 31%). Indeed, the average DNA methylation levels of the 3223 DMCpG sites were greater in post-surgery patients (0.49 ± 0.18 versus 0.45 ± 0.19, *p* < 0.001).

### Identification of target genes specifically associated with body weight changes

**Fig. 1:**
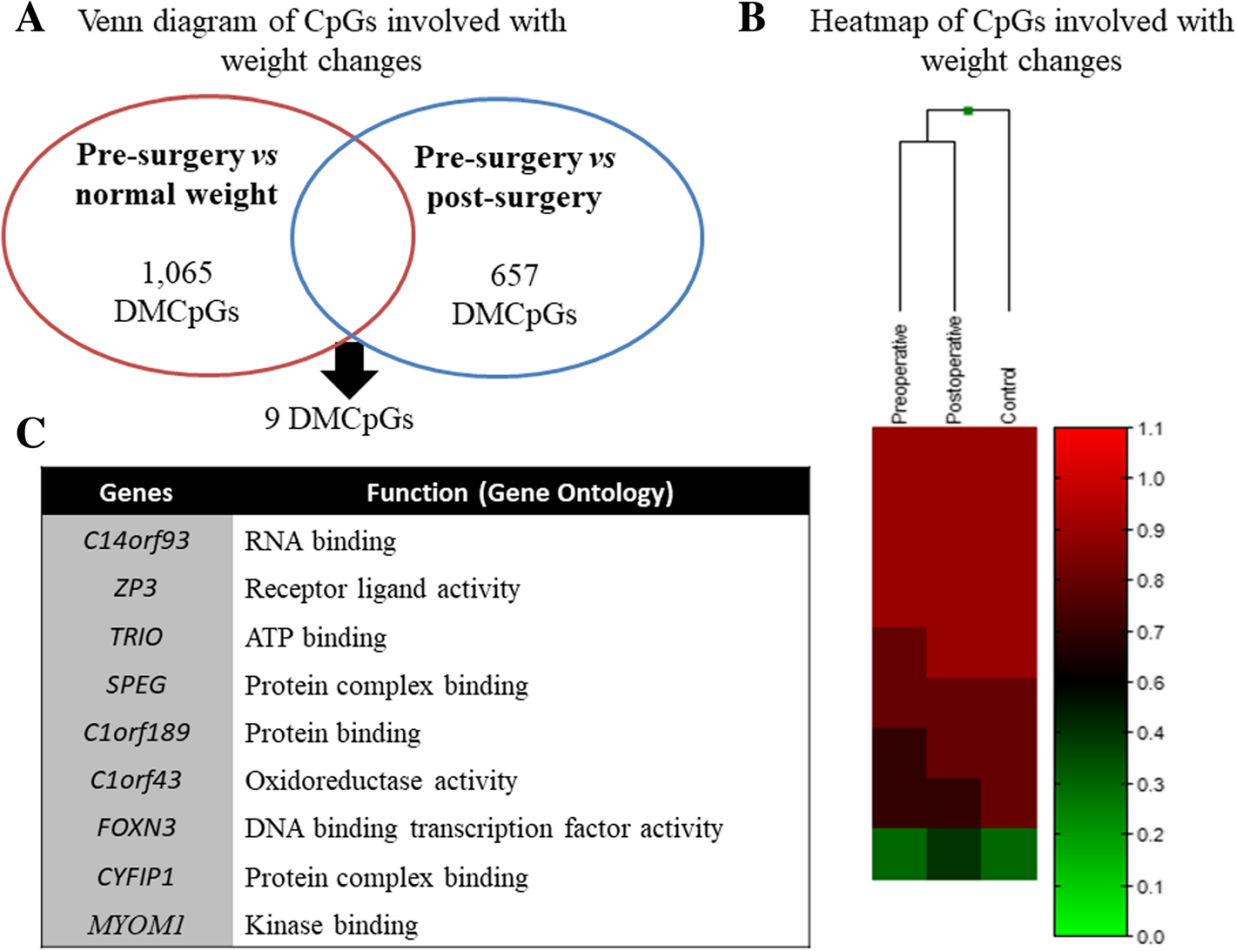
Clustering analysis of the differentially methylated CpG sites. **a**: Venn diagram of the DMCpG sites detected in the pre-surgery patients versus the controls and in the pre-surgery period versus the post-surgery period. Nine common sites indicated genes related to body weight changes. **b**: Supervised clustering of the nine CpGs that were differentially methylated in the pre-surgery patients as compered to the controls and which had their methylation profiles altered after the surgical procedure. **c**: Gene function of eight genes represented by the nine differentially methylated CpG sites

Comparison between DMCpG sites in the pre-surgery patients versus the controls and in the pre-surgery period versus the post-surgery period by means of a Venn diagram showed that nine CpGs located in nine different genes were common in both analyses (Fig. 1a). These CpGs exhibited lower methylation levels in the pre-surgery e patients as compared to the controls, after RYGB, the methylation levels increased and neared the levels verified in the controls (Fig. 1b). Table 1 lists the nine CpGs associated with body weight changes as revealed by this study. The largest difference was observed for cg04789056, located in the chromosome 14 open reading frame 93 (*C14orf93*) gene (− 13% in the pre-surgery patients as compared to the controls). This was also the CpG that changed the most after RYGB (+ 13% in the post-surgery period as compared to the preoperative period). Figure 1c indicates the gene functions, which include DNA and RNA binding, protein complex binding, and receptor ligand activity.

**Table 1:** CpG sites which were differently methylated between obese patients before the Roux-en Y gastric bypass and normal weight women and changed with surgical procedure

TargetID	CHR	Gene name	Gene region	Gene context	Methylation level normal weight	Methylation level pre-surgery	Methylation level post-surgery	Δ*	p*	Δ**	p**
cg04789056	14	*C14orf93*;*C14orf93*;*C14orf93*	3’UTR;3’UTR;3’UTR	S_Shelf	0.90 ± 0.02	0.76 ± 0.03	0.89 ± 0.03	−0.13	0.0013	0.13	0.0032
cg01219670	7	*ZP3*;*ZP3*	Body;Body		0.79 ± 0.02	0.69 ± 0.07	0.76 ± 0.05	−0.10	0.0003	0.07	0.0009
cg05590156	5	*TRIO*	Body		0.81 ± 0.02	0.71 ± 0.07	0.77 ± 0.06	−0.10	0.0003	0.06	0.0020
cg02747563	7			N_Shelf	0.94 ± 0.02	0.87 ± 0.08	0.92 ± 0.08	−0.07	0.0093	0.05	0.0011
cg03030650	2	*SPEG*	Body	S_Shelf	0.95 ± 0.01	0.90 ± 0.02	0.95 ± 0.01	−0.05	0.0019	0.05	0.0046
cg04934776	1	*C1orf189*;*C1orf43*;*C1orf43;C1orf43*	TSS1500;3’UTR;3’UTR;3’UTR		0.88 ± 0.01	0.82 ± 0.02	0.88 ± 0.02	−0.05	0.0036	0.05	0.0004
cg03441544	15	*CYFIP1*;*CYFIP1*	Body;Body		0.93 ± 0.01	0.87 ± 0.02	0.93 ± 0.01	−0.05	0.0029	0.05	0.0014
cg02212698	14	*FOXN3*;*FOXN3*	3’UTR;3’UTR	N_Shore	0.96 ± 0.01	0.91 ± 0.01	0.97 ± 0.01	−0.05	0.0013	0.05	0.0002
cg05486872	18	*MYOM1*;*MYOM1*	3’UTR;3’UTR	N_Shore	0.33 ± 0.07	0.40 ± 0.08	0.47 ± 0.11	0.07	0.0080	0.08	0.0008

Values showed in mean ± standard deviation; CHR: chromosome; Δ*: variation between pre-surgery patients and normal weight women; Δ**: variation between pre-surgery and post-surgery patients; p*: comparing pre-surgery patients and normal weight women; p**: comparing pre-surgery and post-surgery patients; UTR: untranslated region; TSS: transcription start site; *C14orf93*: chromosome 14 open reading frame 93; *ZP3*: zona pellucida glycoprotein 3; *TRIO*: trio Rho guanine nucleotide exchange factor; *SPEG*: SPEG Complex Locus; *C1orf189*: chromosome 1 open reading frame 189; *C1orf43*: chromosome 1 open reading frame 43; *CYFIP1*: cytoplasmic FMR1 interacting protein 1; *FOXN3*: forkhead box N3; *MYOM1*: myomesin 1

### Identification of genes specific for obesity status

**Fig. 2:**
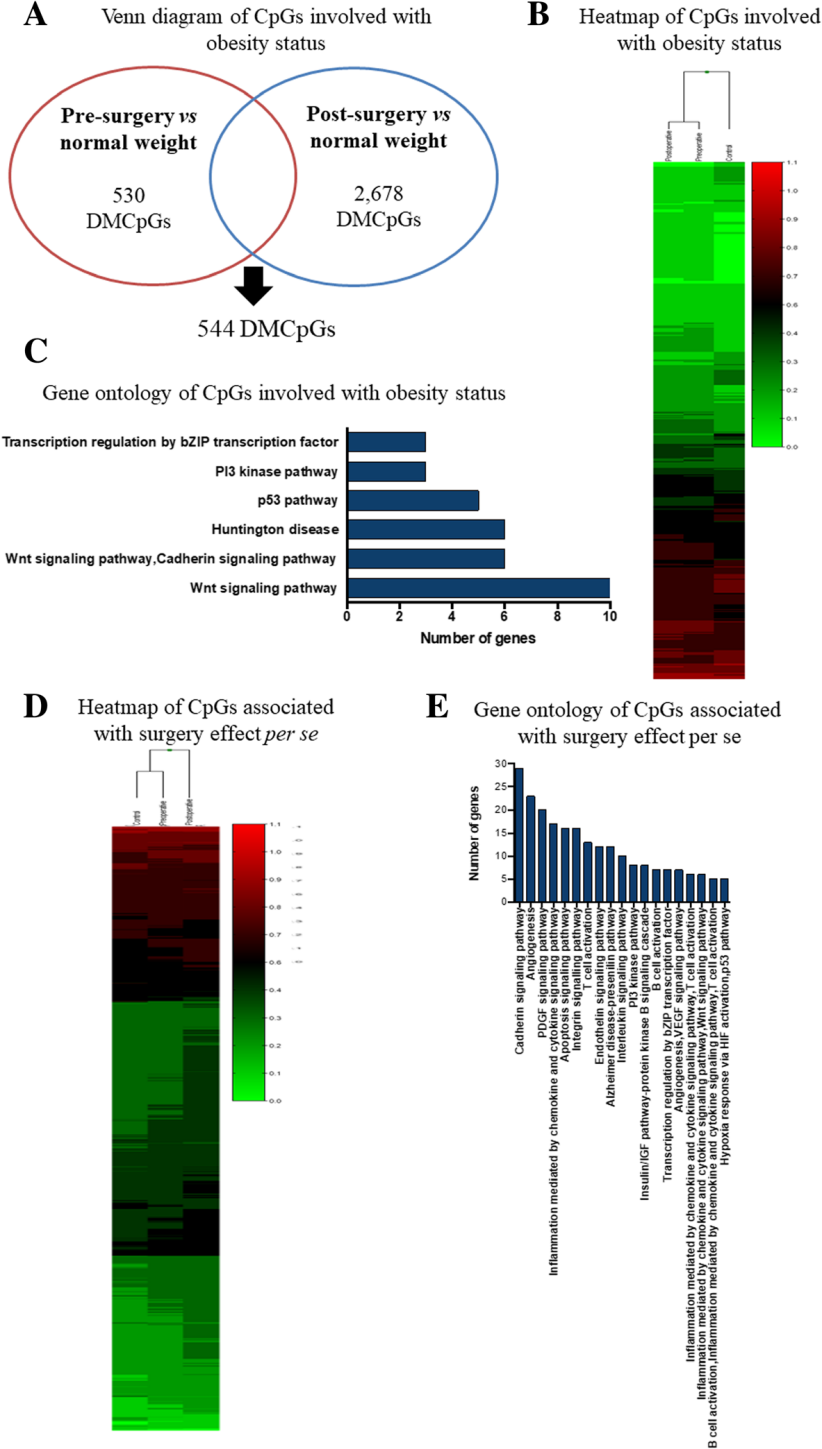
Clustering analysis of the differentially methylated CpG sites. **a**: Venn diagram of the DMCpG sites in the pre-surgery patients as compared to the controls and in the post-surgery patients as compared to the controls. A total of 544 common sites were associated with obesity status, and 2678 DMCpG sites were related to an effect of bariatric surgery per se. **b**: Supervised clustering of the 544 CpGs that were differentially methylated in the pre-surgery patients as compared to the controls. **c**: Summary of the gene ontology (GO) analysis of the biological process categories for the 386 genes represented by the 544 differentially methylated CpG sites. **d**: Supervised clustering of the 2678 CpGs that were differentially methylated in the post-surgery patients as compared to the controls. **e**: Summary of the gene ontology (GO) analysis of the biological process categories for the 1638 genes represented by the 2678 differentially methylated CpG sites

The Venn diagram depicting the DMCpG sites found in the pre-surgery patients versus the controls and in the post-surgery period versus the pre-surgery period showed that the severely obese patients and the controls always had 544 different sites, located in 386 unique genes (Fig. 2a). These CpG sites were different in the controls and were not influenced by the surgical procedure (Fig. 2b). Table 2 summarizes the top 20 CpG sites, which were always differentially methylated in the severely obese patients as compared to the controls. Among the encoded genes, NADH:ubiquinone oxidoreductase subunit S6 (*NDUFS6*) and mitochondrial ribosomal protein L36 (*MRPL36*) were the genes that were the most represented with two DMCpG sites located in the island.

**Table 2:** Top 20 CpG sites which were differently methylated between obese patients and normal weight women and did not change after bariatric surgery

TargetID	Gene name	CHR	Gene region	Gene context	Methylation level normal weight	Methylation level Pre-surgery	Methylation level Post-surgery	Δ*	p*	Δ**	p**
*Hypomethylated CpG sites in obese women*											
cg01428687	*C3orf10*	3	TSS1500	N_Shore	0.59 ± 0.04	0.31 ± 0.23	0.32 ± 0.24	−0.27	<0.0001	−0.27	0.0003
cg05926253	*NDUFS6*;*MRPL36*	5	TSS200;TSS1500	Island	0.58 ± 0.03	0.32 ± 0.27	0.31 ± 0.27	−0.26	0.0013	−0.27	0.0003
cg07402800	*FBXL6*;*GPR172A*;*FBXL6*	8	1stExon;TSS1500;1stExon	Island	0.75 ± 0.02	0.54 ± 0.20	0.55 ± 0.22	−0.20	0.0005	−0.20	0.0045
cg04118610	*LPHN3*	4	Body		0.59 ± 0.20	0.39 ± 0.27	0.38 ± 0.26	−0.20	0.0058	−0.21	0.0031
cg06002203	*TNXB*;*TNXA*	6	Body;Body		0.82 ± 0.03	0.66 ± 0.27	0.67 ± 0.26	−0.16	0.0005	−0.15	0.0014
cg04427462	*PRKD2*;*PRKD2*;*PRKD2*;*PRKD2*	19	TSS1500;Body;Body;Bod.	N_Shore	0.49 ± 0.02	0.34 ± 0.15	0.35 ± 0.16	−0.15	0.0006	−0.15	0.0026
cg02688348	*SSRP1*	11	TS200	Island	0.41 ± 0.02	0.26 ± 0.15	0.26 ± 0.15	−0.15	0.0006	−0.15	0.0002
cg02037503	*ACIN1*;*ACIN1*;*ACIN1*;*ACIN1*;*ACIN1*;*ACIN1*	14	1stExon;1stExon;Body;Body;Body;5’UTR		0.52 ± 0.01	0.37 ± 0.15	0.36 ± 0.15	− 0.14	0.0009	−0.15	0.0002
cg00973947	*C3orf58*;*C3orf58*;*C3orf58*	3	5’UTR;Body;1stExon	Island	0.40 ± 0.03	0.25 ± 0.14	0.25 ± 0.14	−0.14	0.0003	−0.15	0.0004
cg04707013		10			0.74 ± 0.05	0.60 ± 0.22	0.56 ± 0.26	−0.14	0.0007	−0.18	0.0012
*Hypermethylated CpG sites in obese women*											
cg00087746		12			0.49 ± 0.25	0.69 ± 0.14	0.69 ± 0.14	0.21	0.0049	0.20	0.0041
cg03071500		11			0.54 ± 0.15	0.69 ± 0.17	0.75 ± 0.13	0.14	0.0027	0.20	0.0004
cg03871140		5		N_Shore	0.19 ± 0.18	0.31 ± 0.22	0.30 ± 0.22	0.12	0.0031	0.10	0.0041
cg00159523	*TCF7L2*;*TCF7L2*;*TCF7L2*;*TCF7L2*;*TCF7L2*;*TCF7L2*	10	Body;Body;Body;Body;Body;Body	S_Shore	0.45 ± 0.11	0.57 ± 0.13	0.60 ± 0.12	0.11	0.0054	0.15	0.0003
cg05226335	*CTTN*;*CTTN*	11	Body;Body	N_Shelf	0.52 ± 0.12	0.63 ± 0.09	0.66 ± 0.10	0.11	0.0017	0.14	0.0002
cg05164937		10		S_Shore	0.36 ± 0.08	0.47 ± 0.08	0.45 ± 0.09	0.11	<0.0001	0.09	0.0006
cg01579765	*HSF2BP*	21	Body	N_Shore	0.19 ± 0.03	0.30 ± 0.09	0.30 ± 0.08	0.11	0.0004	0.11	0.0006
cg08206623	*CDKN1C*;*CDKN1C*;*CDKN1C*	11	TSS1500;TSS1500;TSS1500	Island	0.35 ± 0.03	0.45 ± 0.09	0.43 ± 0.08	0.11	0.0010	0.08	0.0054
cg04352272		17		Island	0.18 ± 0.07	0.28 ± 0.09	0.26 ± 0.09	0.10	0.0011	0.09	0.0026
cg01357671	*ITGAE*;*GSG2*	17	Body;TSS1500	N_Shore	0.15 ± 0.02	0.25 ± 0.10	0.23 ± 0.10	0.10	0.0004	0.08	0.0018

Values showed in mean ± standard deviation; CHR: chromosome; Δ*: variation between pre-surgery patients and normal weight women; Δ**: variation between post-surgery patients and normal weight women; p*: comparing pre-surgery patients and normal weight women; p**: comparing post-surgery patients and normal weight women; UTR: untranslated region; TSS: transcription start site; *NDUFS6*: NADH ubiquinone oxidoreductase subunit S6; *MRPL36*: mitochondrial ribosomal protein L36; *FBXL6*: F-box and leucine rich repeat protein 6; *GPR172A*: solute carrier family 52 member 2; *LPHN3:* adhesion G protein-coupled receptor L3; *TNXB*: tenascin XB; *TNXA*: tenascin XA; *PRKD2:* protein kinase D2; *SSRP1*: structure specific recognition protein 1; *ACIN1:* apoptotic chromatin condensation inducer 1; *C3orf58:* chromosome 3 open reading frame 58; *TCF7L2:* transcription factor 7 like 2; *CTTN:* cortactin; *HSF2BP:* heat shock transcription factor 2 binding protein; *CDKN1C:* cyclin dependent kinase inhibitor 1C; *ITGAE:* integrin subunit alpha E

GO analysis helped to investigate the potential biological relevance of the genes with different DNA methylation status in the severely obese patients and the controls (Fig. 2c). Regarding biological processes, most of the differentially methylated genes were associated with transcription regulation, signal transduction, apoptosis, transport, and cell adhesion. Interestingly, pathway analysis identified that most of the genes were related to the Wnt and p53 signaling pathways (Fig. 2c).

### Identification of genes related to the effect of bariatric surgery per se

According to evidence gathered herein, 2678 CpG sites were not statistically different in the severely obese patients and the controls, however, these genes became differentially methylated after RYGB. These DMCpG sites were located in 1638 genes, most of the sites (2219 CpGs) showed high methylation after RYGB (Fig. 2d). Table 3 depicts the top 20 CpG sites that were differently methylated in the post-surgery patients as compared to the controls. The most significant difference was observed for cg07875360 in the *NDUFS6* and *MRPL36* genes (+ 35% in the controls as compared to the post-surgery patients).

**Table 3:** Top 20 CpG sites that became differently methylated from control group after Roux-en Y gastric bypass

TargetID	Gene name	CHR	Gene region	Gene context	Methylation level normal weight	Methylation level Post-surgery	Δ	*p*
*Hypomethylated CpG sites in obese women after RYGB*								
cg07875360	*NDUFS6*;*MRPL36*	5	TSS200;TSS1500	Island	0.72 ± 0.03	0.40 ± 0.32	−0.31	0.0011
cg01759136		6			0.68 ± 0.04	0.40 ± 0.30	−0.28	0.0034
cg00554442	*LMF1*	16	TSS200	Island	0.26 ± 0.01	0.01 ± 0.01	−0.25	0.0046
cg05444312	*HIST1H2BM*;*HIST1H2AJ*	6	TSS200;TSS200	S_Shore	0.40 ± 0.04	0.21 ± 0.19	−0.19	0.0009
cg07962043	*TMEM132A*;*TMEM132A*	11	TSS1500;TSS1500	N_Shore	0.38 ± 0.02	0.25 ± 0.11	−0.13	<0.0001
cg04450994	*SLC22A23*;*SLC22A23*	6	Body;Body		0.56 ± 0.06	0.45 ± 0.08	−0.11	0.0003
cg01035945	*ZNF323*;*ZNF323*;*ZKSCAN3*	6	5’UTR;5’UTR;TSS1500		0.25 ± 0.03	0.14 ± 0.12	−0.11	0.0054
cg07929642	*ANKRD11*	16	5’UTR		0.75 ± 0.05	0.64 ± 0.08	−0.11	0.0006
cg06115576	*DIP2C*	10	Body		0.86 ± 0.02	0.75 ± 0.18	−0.11	<0.0001
cg00510787	*C6orf211*;*RMND1*	6	TSS1500;5’UTR	N_Shore	0.29 ± 0.03	0.18 ± 0.13	−0.11	0.0097
*Hypermethylated CpG sites in obese women after RYGB*								
cg07275179	*ATXN7*;*ATXN7*	3	Body;Body		0.41 ± 0.09	0.53 ± 0.11	0.12	0.0002
cg07212327	*FAM49B*	8	5’UTR	N_Shelf	0.50 ± 0.09	0.62 ± 0.10	0.12	<0.0001
cg07401324	*PTPRJ*;*PTPRJ*	11	Body;Body		0.35 ± 0.07	0.48 ± 0.09	0.12	0.0002
cg02353916	*LOC285550*	4	3’UTR		0.44 ± 0.10	0.57 ± 0.10	0.13	<0.0001
cg00123214	*RWDD3*;*RWDD3*	1	Body;Body	S_Shelf	0.52 ± 0.23	0.65 ± 0.15	0.13	0.0058
cg02387226	*UNG*;*UNG*	12	TSS1500;TSS1500	N_Shore	0.52 ± 0.02	0.65 ± 0.03	0.13	0.0069
cg03494429		12			0.64 ± 0.07	0.79 ± 0.06	0.14	0.0093
cg04412904		16			0.22 ± 0.19	0.37 ± 0.23	0.15	0.0028
cg07093060		3		N_Shelf	0.66 ± 0.29	0.84 ± 0.10	0.18	0.0082
cg07572984		14			0.60 ± 0.30	0.80 ± 0.20	0.20	0.0013

Values showed in mean ± standard deviation; CHR: chromosome; Δ: variation between post-surgery patients and normal weight women; *p*: comparing post-surgery patients and normal weight women UTR: untranslated region; TSS: transcription start site; *NDUFS6:* NADH:ubiquinone oxidoreductase subunit S6; *MRPL36*: mitochondrial ribosomal protein L36; *LMF1*: lipase maturation factor 1; *HIST1H2BM*: histone cluster 1 H2B family member m; *HIST1H2AJ*: histone cluster 1 H2A family member j; *TMEM132A*: transmembrane protein 132A; *SLC22A23*: solute carrier family 22 member 23; *ZNF323*: zinc finger and SCAN domain containing 31; *AN*3: tripartite motif containing 44; *ANKRD11*: ankyrin repeat domain 11; *DIP2C*: disco interacting protein 2 homolog C; *C6orf211*: acidic residue methyltransferase 1; *RMND1*: required for meiotic nuclear division 1 homolog; *ATXN7*: ataxin 7; *FAM49B*: family with sequence similarity 49 member B; *PTPRJ*: protein tyrosine phosphatase, receptor type J; *RWDD3*: RWD domain containing 3; *UNG*: uracil DNA glycosylase

GO analysis helped to evaluate the biological processes and pathways of the genes with DMCpG sites in the post-surgery patients and the controls. Among the biological processes, transcription regulation, signal transduction, cell adhesion, blood coagulation, apoptotic process, ion transport, and protein phosphorylation exhibited the majority of genes. Indeed, pathway analysis identified that the genes were related to cadherin signaling, angiogenesis, apoptosis, inflammation, and interleukin pathways (Fig. 2e).

### Epigenetic signatures and phenotypic characteristics

**Fig. 3:**
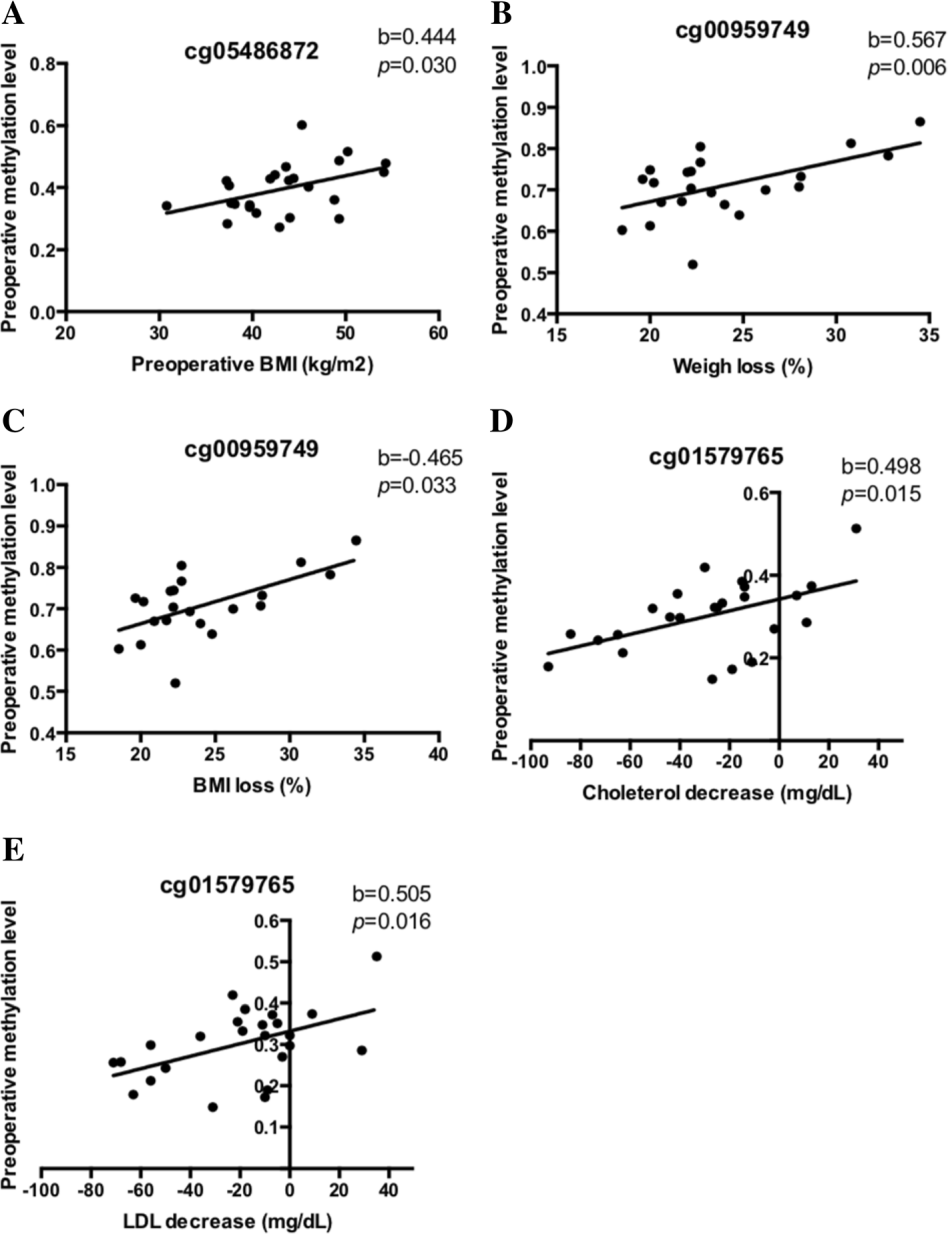
Linear regression models adjusted by age showing the positive effect of baseline DNA methylation on the phenotypic characteristics. **a** Effect of the MYOM1 gene on BMI. **b** Effect of the TMEM48 gene on the percentage of weight loss. **c** Effect of the TMEM48 gene on the percentage of BMI change. **d** Effect of the HSF2BPgene on cholesterol decrease. **e** Effect of the HSF2BP gene on LDL-cholesterol decrease. BMI: body mass index. LDL: low-density lipoprotein

Among the nine genes related to body weight changes and the top 20 CpG sites associated with obesity status, the methylation levels of the myomesin 1 (*MYOM1*), transmembrane protein 48 (*TMEM48*), and heat shock transcription factor 2 binding protein (*HSF2BP*) genes were associated with the anthropometric and biochemical features (Fig. 3). Pre-surgery patients with higher cg05486872 methylation (located in the *MYON1* gene) had greater BMI. Moreover, cg00959749 located in the *TMEM48* gene was always differentially methylated in the severely obese patients as compared to the controls, which indicated that this methylation was positively correlated (baseline level) with the percentage of weight loss and BMI change. This CpG site presented low methylation levels in the severely obese patients. The patients with greater methylation levels at this site lost more weight. Another CpG was also associated with changes in lipid profile after the surgical procedure: cg01579765 in the *HSF2BP* gene was positively correlated with reduced cholesterol and LDL concentrations. The effect of methylation levels on these phenotypic characteristics remained apparent after regression adjusted by age.

## Discussion

Genome-wide DNA methylation analysis identified CpG sites that are specific for obesity per se after bariatric surgery, the identified sites remained different in the operated patients as compared to the controls. This analysis also identified CpG sites that are modified by bariatric surgery-induced weight loss as well as CpG sites that have their methylation levels specifically modified by RYGB irrespective of their association with the obesity status.

The obesity pathophysiology is strongly associated with adipose tissue dysregulation and epigenetic changes are more tissue specific. This fact represents a huge challenge to evaluate epigenetic mechanisms in longitudinal studies because adipose tissue is an inaccessible tissue without surgery. Instead of adipose tissue biopsies, peripheral blood cells are frequently used for epigenetic analysis. In this regard, very recently it was demonstrated that epigenetic biomarkers in blood can mirror epigenetic signatures in biologically relevant target tissues such as adipose tissue [26–28]. These previous results suggest that the assessment of DNA methylation in whole blood can identify robust and biologically relevant epigenetic variation. Strikingly, we were able to identify epigenetic markers associated with adipose tissue and inflammation (obesity pathogenesis) in blood leukocytes. The identified epigenetic signature could be relevant to the personalized management of obesity, mainly after bariatric surgery, aiming for better result of weight loss.

The scientific literature contains extensive description of the advantages of bariatric surgery over acute significant weight loss in terms of the improvement in comorbidities [29, 30]. Nevertheless, little is known about the epigenetic mechanisms associated with the metabolic and clinical benefits provided by bariatric surgery. The present study showed that RYGB promoted changes in 666 CpG sites. Previous evidence suggests that changes in whole blood DNA methylation may be related to body weight and fasting plasma glucose reduction [31]. Other studies have described epigenetic changes in the adipose tissue [32, 33] and skeletal muscle [34] after RYGB.

In this context, we were able to identify 544 DMCpG sites related to obesity status in leukocytes. Interestingly, these sites remained differentially methylated in obese and normal-weight women irrespective of the bariatric surgery effect on weight loss. The main routes associated with these sites were the Wnt and p53 signaling pathways, which are associated with adipocyte differentiation [35, 36] and lipid/insulin resistance metabolism [37, 38], respectively. We hypothesized that the weight loss reached by the operated patients six months after surgery was not able to modify this epigenetic profile because the patients remained obese at this time. Based on our analysis, only nine CpG sites were specifically related to body weight changes, which highlighted genes involved in obesity, adipogenesis, and hepatic glucose utilization. These data suggested that the genes associated with these CpG sites should be investigated as new and specific targets of obesity and weight loss in future studies.

On the other hand, bariatric surgery promoted epigenetic regulation of several genes, which seemed to be an effect of the surgery per se because their differential methylation was not associated with the obesity status. These CpG sites encoded mainly genes related to angiogenesis, inflammation, and endothelin-signaling pathways. This could be explained by the fact that the surgery itself was an invasive procedure that promoted cell damage and inflammation, with consequent tissue regeneration. Moreover, our previous findings had shown distinct changes in the methylation profile of inflammatory genes after different obesity treatments, with reduction in the *IL-6* methylation level six months after RYGB [18].

Little is discussed about the effects that the different surgical techniques may have on the DNA methylation profile. Knowing that biochemical (bile acids, cholesterol, glycemia) and metabolic (ie improvement of insulin resistance, dyslipidemia, inflammation, oxidative stress) changes due to bariatric surgery may alter the DNA methylation profile of several genes [39] and that the different surgical techniques (restrictive, disabsorptive or mixed) cause diverse nutritional and metabolic changes [40] it is of great value the comparison of the bariatric techniques. In this context, authors found no difference in the methylation of the interspersed nucleotide element 1 (*LINE-1*) in patients underwent Roux-en-Y gastric bypass or laparoscopic sleeve gastrectomy [41].

Nowadays researches are made in the identification of specific biomarkers predicting the response to RYGB procedure [42]. As an example, a previous study by our research group identified difference between the baseline *SERPINE-1* methylation of the individual who lost more or less weight after bariatric surgery [43]. On the other hand, other authors recently found no association between the methylation level of food intake-related genes and the response to surgery [42]. In line of this, linear regression analysis between the baseline methylation levels and the phenotypic markers were performed and showed association of the *MYOM1*, *TMEM48*, and *HSF2BP* methylation levels with the anthropometric and metabolic parameters. Despite the scarce literature data on this topic, different *MYOM1* gene expression has been detected in human skeletal muscle cells of obese and lean subjects [44]. Furthermore, a study evaluating the adipose tissue of obese patients submitted to a six-month caloric restriction intervention showed different *TMEM48* expression between high and low responders to dieting [19].

Studies on epigenetic patterns have become highly important due to their plasticity in the face of external factors and to the individual’s own response. Better understanding of the pathways altered by bariatric surgery will aid the development of new biomarkers and therapies for obesity treatment [39, 45].

The strength of this study lies on its longitudinal design, which allowed us to evaluate whether changes in the methylation profiles were due to surgical intervention and/or body weight loss. Although the observed differences were statistically significant, the magnitude of DNA methylation differences between the pre- and the post-surgery periods may be considered small. A possible explanation for this small magnitude would be the reduced period of postoperative evaluation.

## Conclusion

RYGB promoted epigenetic changes in specific pathways, mainly the pathways related to inflammation, angiogenesis, and endothelin-signaling. Genome-wide DNA methylation analysis revealed that gene clusters remained unchanged even after bariatric surgery, suggesting that, despite the strong magnitude of the weight loss achieved by the patients 6 months after bariatric surgery, reaching a normal body weight may be necessary to revert the methylation profile associated with obesity.

## Additional files

Additional file 1: Table. Anthropometry, body composition and biochemical data of women who underwent Roux in Y gastric bypass and normal weight controls. (DOCX 19 kb)

Additional file 2: Figure. Gene regions of differential methylated CpG sites. (DOCX 199 kb)

Additional file 3: Figure. Diagram of differential methylated CpG sites founded in present study. (TIF 58 kb)

Additional file 4: Raw data. Datasets generated and/or analyzed during the current study. (XLSB 395235 kb)

## Abbreviations

BMI: Body Mass Index
DMCpGs: Differentially methylated CpG sites
GO: Gene ontology
RYGB: Roux-en Y gastric bypass
TSS: Transcription start site
UTR: Untranslated region
WC: Waist circumference
