# Supplementary material for: DNA methylation screening after roux-en Y gastric bypass reveals the epigenetic signature stems from genes related to the surgery per se
Source: BMC Med Genomics. 2019 May 27;12:72. doi: 10.1186/s12920-019-0522-7 (PMC6537208; doi:10.1186/s12920-019-0522-7)
Supplement: Supplementary file 1 — Table. Anthropometry, body composition and biochemical data of women who underwent Roux in Y gastric bypass and normal weight controls. (DOCX 19 kb) [file 12920_2019_522_MOESM1_ESM.docx]

| Supplementary 1. Anthropometry, body composition and biochemical data of women who underwent Roux in Y gastric bypass and normal weight controls | | | | | | |
| --- | --- | --- | --- | --- | --- | --- |
| Variables | Normal weight (n=24) | Pre-surgery  (n=24) | Post-surgery  (n=24) | *p** | *p*** | *p**** |
| Weight (kg) | 60.7±6.3 | 113.3±16.3 | 86.3±12.5 | <0.001 | <0.001 | <0.001 |
| Height (m) | 1.64±0.06 | 1.61±0.08 | - | 1.00 | - | - |
| BMI (kg/m²) | 22.5±1.6 | 43.3±5.7 | 33.1±4.8 | <0.001 | <0.001 | <0.001 |
| WC (cm) | 78.7±7.2 | 124.7±14.9 | 107.0±13.5 | <0.001 | <0.001 | <0.001 |
| FFM (%) | 70.4±4.1 | 54.1±6.1 | 55±3.8 | <0.001 | <0.001 | <0.001 |
| FM (%) | 29.6±4.1 | 45.7±5.7 | 36.1±5.9 | <0.001 | <0.001 | <0.001 |
| Glucose (g/dL) | 81.2±8.8 | 96.1±28.2 | 83.6±6.7 | 0.037 | 0.024 | 0.321 |
| TC (g/dL) | 171.6±31.4 | 183.3±37.3 | 154.2±32.9 | 0.290 | <0.001 | 0.091 |
| LDL-c (g/dL) | 100.8±27.4 | 111.7±33.9 | 91.1±25.1 | 0.269 | 0.002 | 0.243 |
| HDL-c (g/dL) | 56.7±13.7 | 44±7.8 | 46.4±10.6 | <0.001 | 0.286 | 0.009 |
| Triglycerides (g/dL) | 70.4±36.7 | 126.8±44.2 | 78.7±22.1 | <0.001 | <0.001 | 0.372 |
| Values showed in mean ± standard deviation; n: number of individuals; BMI: body mass index; WC: waist circumference; FFM: fat free mass; FM: fat mass; TC: Total cholesterol; LDL-c: low density lipoprotein; HDL-c; high density lipoprotein; *p**: comparing pre-surgery and normal weight women; *p***: comparing pre and post-surgery time; *p****: comparing post-surgery and normal weight women | | | | | | |
