# Supplementary figures and images for: DNA methylation screening after roux-en Y gastric bypass reveals the epigenetic signature stems from genes related to the surgery per se
Source: BMC Med Genomics. 2019 May 27;12:72. doi: 10.1186/s12920-019-0522-7 (PMC6537208; doi:10.1186/s12920-019-0522-7)

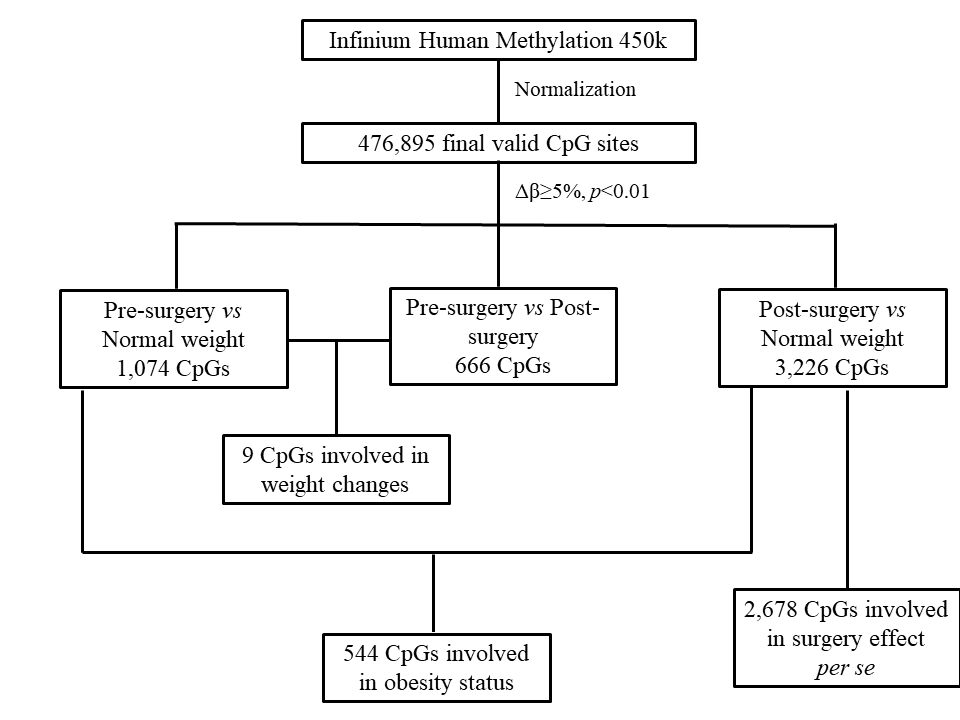

Supplement: Supplementary file 3 — Figure. Diagram of differential methylated CpG sites founded in present study. (TIF 58 kb) [file 12920_2019_522_MOESM3_ESM.tif]
