# Supplementary material for: DNA methylation screening after roux-en Y gastric bypass reveals the epigenetic signature stems from genes related to the surgery per se
Source: BMC Med Genomics. 2019 May 27;12:72. doi: 10.1186/s12920-019-0522-7 (PMC6537208; doi:10.1186/s12920-019-0522-7)
Supplement: Supplementary file 3 [file 12920_2019_522_MOESM2_ESM.docx]

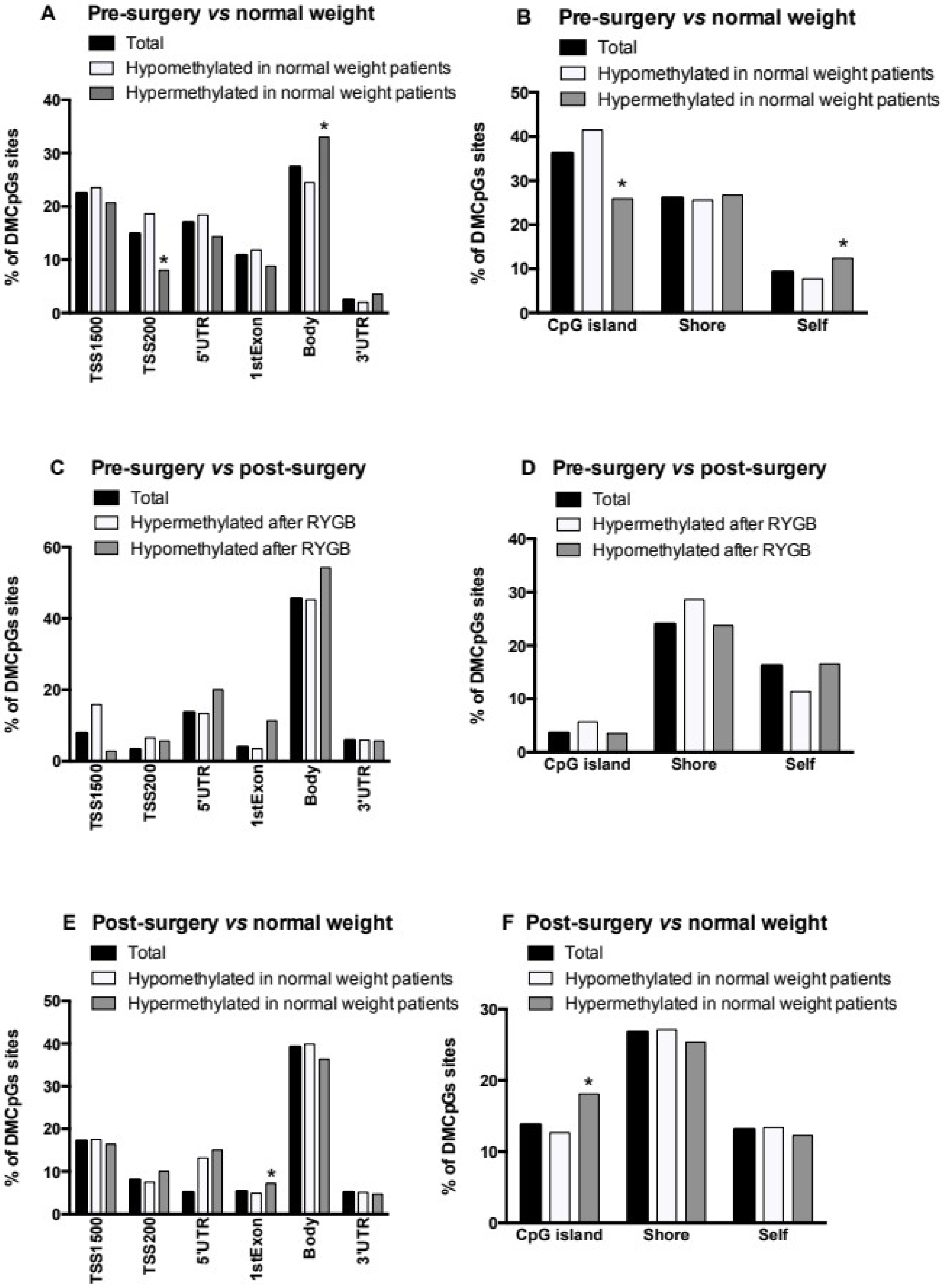
A
Pre-surgery vs normal weight
Total
Hypomethylated in normal weight patients
Hypermethylated in normal weight patients
% of DMCpGs sites
0
10
20
30
40
TSS1500
TSS200
5'UTR
1stExon
Body
3'UTR
*
*
B
Pre-surgery vs normal weight
Total
Hypomethylated in normal weight patients
Hypermethylated in normal weight patients
% of DMCpGs sites
0
10
20
30
40
50
CpG island
Shore
Self
*
*
C
Pre-surgery vs post-surgery
Total
Hypermethylated after RYGB
Hypomethylated after RYGB
% of DMCpGs sites
0
20
40
60
TSS1500
TSS200
5'UTR
1stExon
Body
3'UTR
D
Pre-surgery vs post-surgery
Total
Hypermethylated after RYGB
Hypomethylated after RYGB
% of DMCpGs sites
0
10
20
30
40
CpG island
Shore
Self
E
Post-surgery vs normal weight
Total
Hypomethylated in normal weight patients
Hypermethylated in normal weight patients
% of DMCpGs sites
0
10
20
30
40
50
TSS1500
TSS200
5'UTR
1stExon
Body
3'UTR
*
F
Post-surgery vs normal weight
Total
Hypomethylated in normal weight patients
Hypermethylated in normal weight patients
% of DMCpGs sites
0
10
20
30
CpG island
Shore
Self
*
